# Methylglyoxal Forms Diverse Mercaptomethylimidazole Crosslinks with Thiol and Guanidine Pairs in Endogenous Metabolites and Proteins

**DOI:** 10.1021/acschembio.1c00553

**Published:** 2021-09-28

**Authors:** John S. Coukos, Raymond E. Moellering

**Affiliations:** Department of Chemistry, The University of Chicago, 929 E 57th Street, Chicago, Illinois 60637, United States

## Abstract

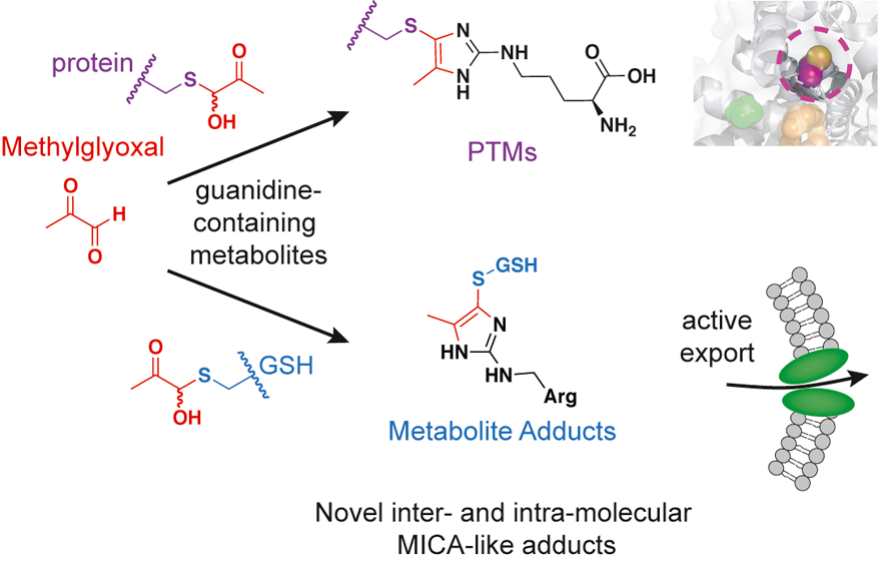

Methylglyoxal (MGO) is a reactive byproduct formed by several metabolic precursors, the most notable being triosephosphates in glycolysis. While many MGO-mediated adducts have been described, the reactivity and specific biomolecular targets of MGO remain incompletely mapped. Based on our recent discovery that MGO can form stable mercaptomethylimidazole crosslinks between cysteine and arginine (MICA) in proteins, we hypothesized that MGO may participate in myriad reactions with biologically relevant guanidines and thiols in proteins, metabolites, and perhaps other biomolecules. Herein, we performed steady-state and kinetic analyses of MGO reactivity with several model thiols, guanidines, and biguanide drugs to establish the plausible and prevalent adducts formed by MGO in proteins, peptides, and abundant cellular metabolites. We identified several novel, stable MICA metabolites that form *in vitro* and in cells, as well as a novel intermolecular post-translational MICA modification of surface cysteines in proteins. These data confirm that kinetic trapping of free MGO by thiols occurs rapidly and can decrease formation of more stable imidazolone (MG-H1) arginine adducts. However, reversible hemithioacetal adducts can go on to form stable MICA modifications in an inter- and intramolecular fashion with abundant or proximal guanidines, respectively. Finally, we discovered that intracellular MICA-glutathione metabolites are recognized and exported by the efflux pump MRP1, providing a parallel and perhaps complementary pathway for MGO detoxification working alongside the glyoxalase pathway. These data provide new insights into the plausible reactions involving MGO in cells and tissues, as well as several new molecular species in proteins and metabolites for further study.

## Introduction

Intrinsically reactive metabolites are known to arise from pools of lipid, glycolytic, citric acid cycle, and respiration-generated intermediates.^1−4^ Central glycolysis in particular harbors several intrinsically reactive metabolites, including the acyl phosphate 1,3-bisphosphoglyceric acid^2,5^ and the highly reactive dicarbonyl methylglyoxal (MGO), which forms from phosphate elimination of the adjacent triosephosphates.^6^ MGO has been found to form a variety of modifications on nucleophilic functional groups in proteins, lipids, and nucleic acids, collectively referred to as advanced glycation end products (AGEs) (Figure S1).^7^ Reversible and stable modifications have been observed on nucleobases within RNA and DNA, which may be associated with altered translation, gene expression, and propensity for mutation.^8,9^ Within proteins, prevalent MGO modifications on arginine residues include hydroimidazolones (MG-H1 and regiochemical isomers), argpyrimidines, and 1-carboxyethyl-arginines (CEA).^10−12^ On the ε-amine of lysine, MGO forms reversible hemiaminals and stable 1-carboxyethyl-lysine (CEL) modifications.^13^ Recently, it has become clear that lactoylglutathione—the first intermediate formed during glyoxalase detoxification of MGO—can modify the ε-amine of lysine in an analogous acylation to other thioesters and mixed anhydrides, forming d-lactoyllysine (Klac).^14^ MGO has also been shown to rapidly form reversible modifications on protein cysteines and small molecule thiols, likely acting as a “depot” of bound-MGO in cells and serum.^15,16^

Despite the fact that many of these modifications were discovered decades ago and that strong links exist between elevated levels of reactive metabolites and disease pathology in humans,^17−19^ the reactivity landscape of MGO remains incompletely mapped. For example, we recently discovered a new methylimidazole modification formed by MGO between proximal cysteine and arginines (MICA) on proteins.^20^ This modification was hypothesized to exist due to the observation that elevated MGO levels in cells resulted in a cysteine-dependent, irreversible homodimerization of the stress-responsive protein KEAP1. Subsequent modeling with peptides suggested that this modification forms *via* reversible hemithioacetal formation on cysteine thiolates, intra- or intermolecular reaction with a proximal arginine, and subsequent dehydration to form a stable mercaptomethylimidazole. Along with other studies, this work confirmed that novel reactivities involving metabolites such as MGO are as yet undiscovered. In particular, there are open questions about the kinetic and thermodynamic reaction sequences of MGO with thiol- and guanidine-containing biomolecules. It is also unclear if MICA-like modifications only form if thiol/guanidine groups are present in close proximity or if significant intermolecular reactivity is possible. Characterizing this reactivity would inform what types of reactive modifications might form in biological systems, providing new target molecules and hypotheses for further study in specific signaling and disease pathology contexts. Here, we demonstrate that diverse mercaptomethylimidazole crosslinks can form between a range of thiol and guanidine containing small-molecule metabolites and drugs, as well as on proteins *in vitro* and in cells. Kinetic characterization of these reaction sequences provides a mechanistic framework to understand the plausible MGO reactions that might occur in cells and tissues. Finally, we demonstrate that several MICA adducts with glutathione and cellular guanidines are actively exported from cells by the multidrug resistance-associate protein MRP1, providing an additional route for MGO detoxification in cells.

## Results and Discussion

### Methylglyoxal Forms Interconnected, Transient, and Stable Modifications with Thiol/Guanidine Pairs

**Figure 1:**
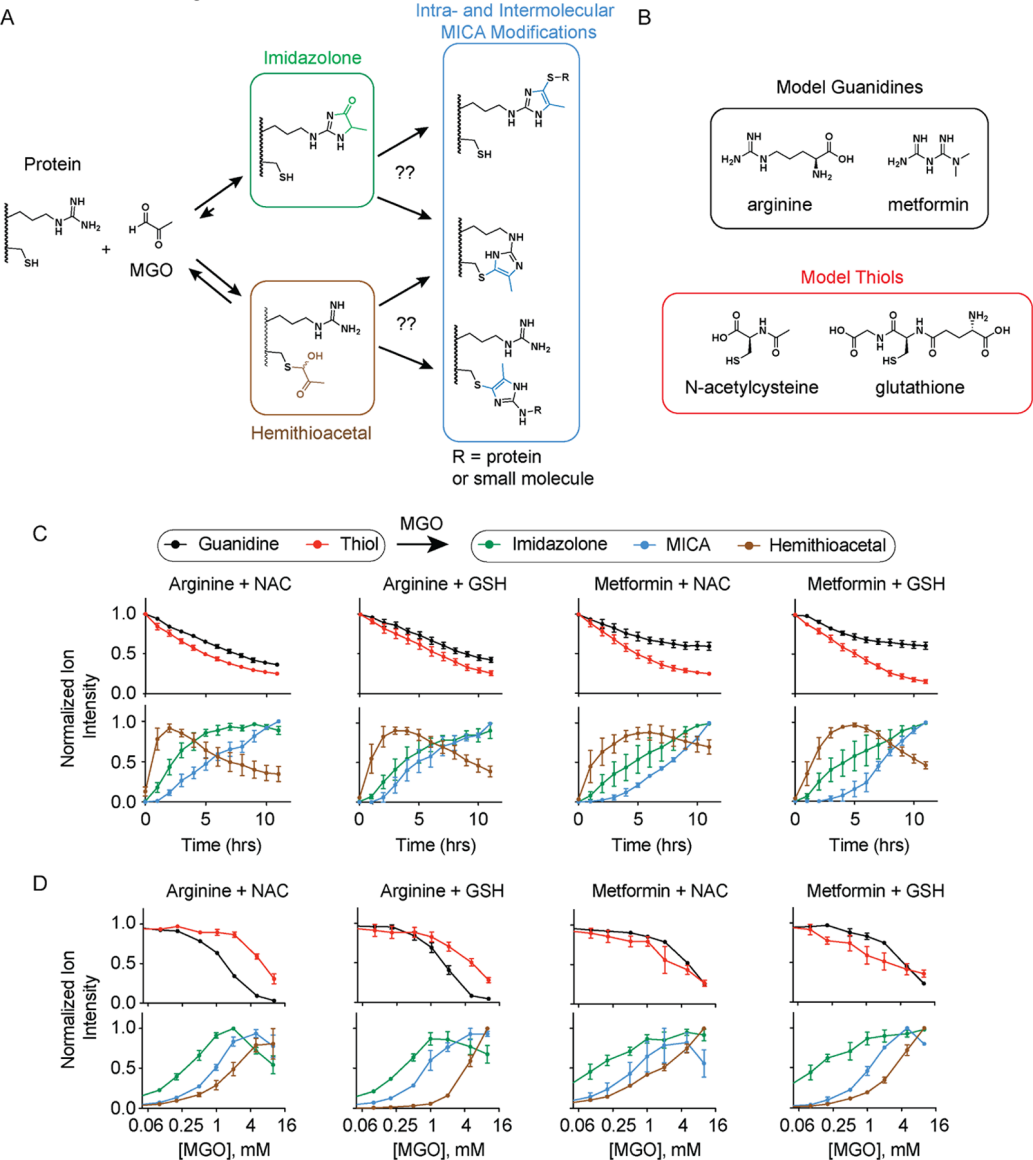
Kinetic and thermodynamic characterization of MGO reactions with biological guanidine and thiols. (A) Schematic depicting known and hypothesized MGO reactivity paths with biologically relevant thiols and guanidine-containing metabolites or proteins. (B) Model guanidine- or thiol-containing metabolites used in this study. (C) LC–MS quantification of indicated metabolite reactants, intermediates, and products within time-course studies of equimolar (1 mM) concentrations of the indicated guanidine, thiol, and MGO at 25 °C. (D) LC–MS quantification of indicated reactant and product levels after 24 h incubation of guanidine and thiol compounds (1 mM each) with indicated MGO concentrations at 37 °C. Data plotted in (C,D) are mean with S.E.M. from *n* = 4 independent biological replicates.

Given the ability for MGO to form stable and reversible modifications on arginine and cysteine in proteins, we speculated that the stable MICA-like crosslinking reaction could occur more broadly between biological thiol- and guanidine-containing molecules in a variety of inter- or intramolecular contexts (Figure 1A). Using liquid chromatography (LC)–mass spectrometry (MS), we monitored the steady-state and kinetic product profiles between MGO and combinations of the model thiols glutathione (GSH) and *N*-acetylcysteine (NAC) and the model guanidines arginine and the drug metformin, chosen due to the observation that metformin reacts with MGO *in vitro* and *in vivo*(21,22) (Figure 1B). In order to determine which thiol–guanidine pairs would react with methylglyoxal to form MICA crosslinks and intermediate adducts, we first analyzed 24 h reactions between MGO and equimolar concentrations of NAC or GSH with arginine or metformin in phosphate-buffered saline (PBS) at 37 °C. We found that methylglyoxal-derived imidazolone, hemithioacetal, and MICA modifications formed in all four reaction pairs (Figure S2). In order to quantify the relative rates of formation of each of the modified species, we monitored the kinetics of each reaction pair at 25 °C with 1 mM of each substrate and MGO (Figure 1C). As expected, we observed more rapid consumption of the thiol-containing reactants than the guanidine-containing reactants, suggesting that thiols are more reactive toward MGO. Likewise, the hemithioacetal MGO products formed more rapidly than the MICA or imidazolone products, consistent with the premise that hemithioacetal products are kinetically favored. After 2–6 h, however, we observed that the level of hemithioacetals began to decrease in all thiol/guanidine pairs, which coincided with increased formation of the imidazolone and MICA products. This is consistent with the notion that the hemithioacetal modification is reversible, whereas the imidazolone and MICA modifications are the thermodynamically favored products. To further explore this conclusion, we performed 24 h reactions at 37 °C with equimolar thiol and guanidine pair concentrations and variable concentrations of MGO, ranging from 0.1 to 10 equiv (Figure 1D). When reactions were given sufficient time to reach equilibrium, we observed that the imidazolone and MICA products reached maximal levels of formation at lower equivalents of MGO than hemithioacetal products. Indeed, we only observed significant accumulation of the hemithioacetal product when multiple equivalents of MGO were added. The rates of guanidine reactant consumption and product formation also suggest that the guanidine-containing arginine is more reactive toward MGO than the biguanide metformin. Taken together, these data help define the multicomponent thiol/guanidine reactivity landscape with MGO, which likely predicts the metabolic products that could form in biologic environments.

### Methylglyoxal Can Form MICA Modifications between Protein Cysteines and Guanidines in Solution

**Figure 2:**
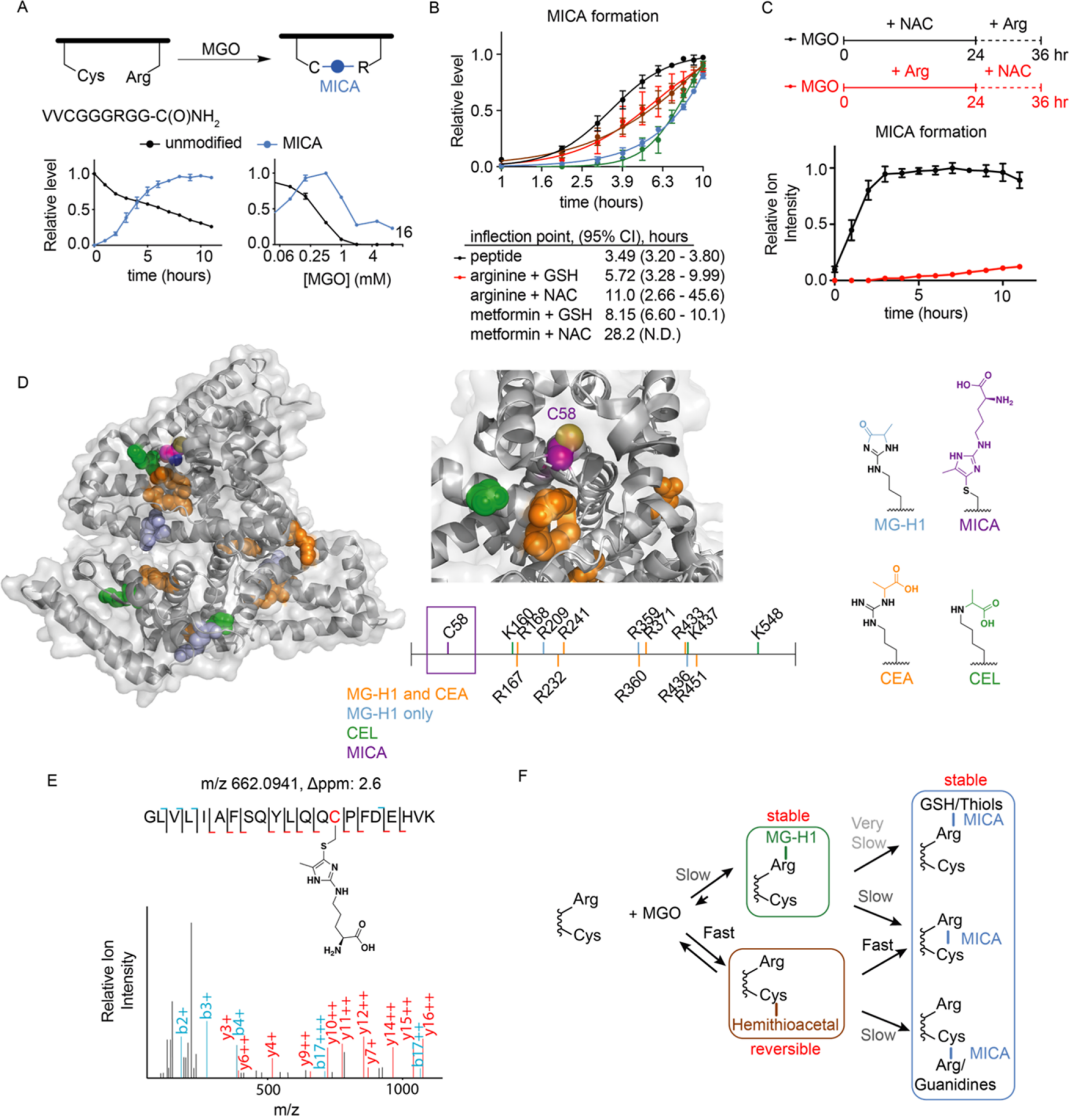
Inter- and intramolecular mercaptomethylimidazole modifications on model peptides and proteins. (A) LC–MS quantification of MGO modifications in the model CRV2 peptide. (B) Logistic fit of MICA formation for peptide time-course experiment and time-course experiments in Figure 1D. (C) LC–MS quantification of MICA product formation under the indicated NAC and Arg cotreatment conditions. (D) Structural depiction (on PDB: 4F5S) of all methylglyoxal-derived modifications observed in BSA treated with MGO and arginine. Chemical structures of each modification are shown (right). (E) Mass spectrum of the Arg-MICA-modified Cys58 peptide detected in BSA. (F) Model summarizing the relative kinetic and thermodynamic landscape of MGO-derived modifications on arginine and cysteine residues. Data plotted in (A–C) represent mean with S.E.M. from independent biological replicates (*n* = 4).

MICA crosslinks may form in either an intermolecular context, for example, between metabolites (Figure 1), or in an intramolecular context. In the context of our previously discovered MICA modification of KEAP1, the high local proximity of Cys151 to Arg135 contributes to efficient formation of the MICA crosslink, allowing the KEAP1-NRF2 pathway to rapidly respond to carbonyl stress.^20^ To assess how local proximity alters MICA modifications on residues within or between proteins, we carried out kinetic and thermodynamic analyses of MGO reactions with a model cysteine- and arginine-containing peptide (Figure 2A). As expected, MICA modification formed more rapidly and at lower equivalents of MGO in the intramolecular context of the peptide than in any corresponding intermolecular reaction between separate thiol- and guanidine- containing molecules (Figure 2A,B). We hypothesized two possible routes of intramolecular MICA crosslink formation based on these data and the intermolecular reaction profiles obtained in Figure 1: (1) initial nucleophilic attack by the thiolate into the aldehyde carbonyl of MGO, followed by attack of the alpha-ketone by the guanidine and subsequent dehydration; (2) initial reaction of the guanidine group with MGO to form a more stable imidazolone, followed by nucleophilic attack of the imidazolone carbonyl by the thiolate and subsequent dehydration (Figure 1A). We directly tested these opposing routes by pre-equilibrating MGO with either NAC or arginine for 24 h, followed by addition of the opposing thiol/guanidine reactant and monitoring for MICA product formation by LC–MS. Reactions with preincubated NAC and MGO, and therefore buildup of the hemithioacetal, formed MICA products much faster and in higher yield than reactions in which the imidazolone was formed first (Figure 2C). Together with the intramolecular reaction monitoring, these data confirm that the predominant route of MICA formation occurs *via* an initial MGO-thiol reaction.

Having confirmed that both intra- and intermolecular MICA modifications form between proximal residues in peptides/proteins and metabolites, respectively, we reasoned that MICA modifications may also form between surface exposed, and perhaps aberrantly reactive cysteines or arginines in proteins. We incubated bovine serum albumin (BSA), which contains a single nondisulfide cysteine and numerous surface arginines with MGO in the presence of soluble arginine or cysteine; these conditions aimed to test whether MICA modifications could form *in trans* on the surface of BSA. High-resolution LC–MS/MS analysis of these protein reactions was then employed to identify MGO-derived post-translational modifications (PTMs). Unsurprisingly, we identified multiple MG-H1 modifications on arginines and more restricted CEA and CEL modifications across the BSA surface (Figure 2D). We did not identify any peptides containing a MICA modification on a surface arginine, however, reinforcing the model that thiol attacks on preformed MG-H1 modified arginines are not a favored route toward mercaptomethylimidazole formation. In contrast, BSA reactions incubated with MGO and arginine in solution contained a single MICA modification site at Cys58, which is the only nondisulfide cysteine in BSA (Figure 2D,E). This cysteine is highly conserved across species, including mouse and human, and is known to form a variety of PTMs, including oxidation and covalent modification by metabolites.^23,24^ Taken together, these data support a model wherein MGO rapidly, yet reversibly, reacts with cysteine residues on the surface of proteins, which may itself transiently affect the protein function or go on to form MICA crosslinks with proximal or abundant guanidine moieties (Figure 2F).

### Several Novel MICA Metabolites Form in Living Cells

**Figure 3:**
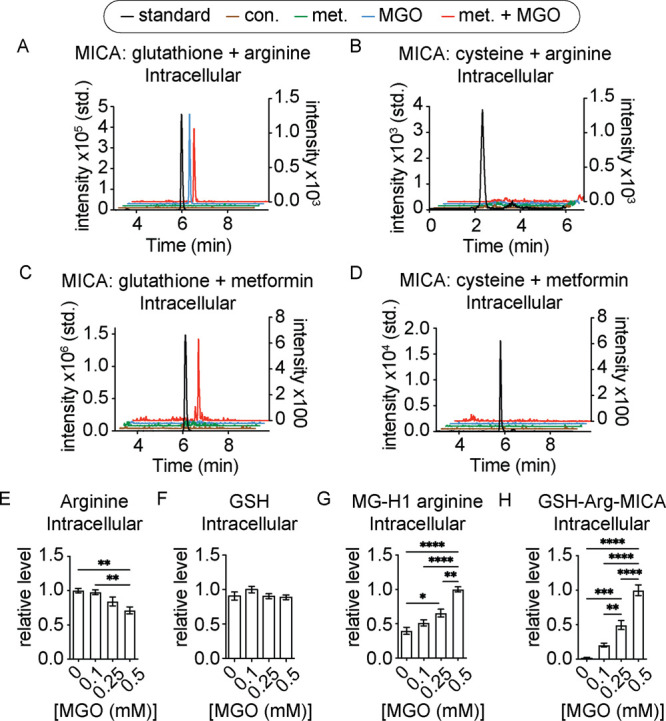
MICA crosslinks form between endogenous thiol/guanidine metabolite pairs in cells. (A–D) Representative chromatograms of MICA cross-linked metabolites between arginine or metformin and glutathione or cysteine in HeLa cells treated with MGO (blue), metformin (green), both (red), or vehicle (brown) for 8 h along with chromatograms of synthetic standards for the relevant MICA adduct (black). (E–H) Relative LC–MS/MS quantification of arginine, glutathione, MG-H1 arginine, and GSH-Arg-MICA in HeLa cells treated with indicated doses of MGO for 8 h. Relative quantification of each metabolite was based off of the condition in which the maximum MS-transition EIC was detected. Data plotted in (E–H) are mean with S.E.M. (*n* = 4 independent biological replicates). Statistical analyses are by ordinary one-way analysis of variance (ANOVA). *p < 0.05; **p < 0.01; ***p < 0.001; ****p < 0.0001.

The model *in vitro* reactivity profiles generated here suggest that several novel MGO-derived metabolites may form in cells. To detect and quantify these species, we first developed mass spectrometry-based, multireaction monitoring (MRM) transitions from synthetic standards for each MICA metabolite (Figure S3). As a first pass to determine whether any or all of the model MICA metabolite species form in cells, we treated HeLa cells with exogenous MGO, with or without physiologically relevant levels of metformin for 8 h and performed targeted metabolomics using the developed transitions. We detected the GSH-Arg-MICA metabolite in cells treated with or without metformin and the GSH-Met-MICA metabolite only in cells treated with metformin (Figure 3A,B). We were unable to detect the MICA metabolite products of metformin or arginine reacting with free cysteine in any of the treatment conditions (Figure 3C,D), which is likely due to the relatively low intracellular concentrations of free cysteine compared to glutathione.^25^ We treated HeLa cells with a range of MGO concentrations up to 0.5 mM, which are consistent with published reports that have reported low-to-mid micromolar concentrations of MGO in various cell types.^26,27^ We found that MGO significantly reduced the detectable levels of free arginine but not glutathione (Figure 3E,F). Consistent with this, we found dose-dependent increases of MG-H1-arginine and the GSH-Arg-MICA metabolite upon MGO treatment (Figure 3G,H). This suggests that arginine availability could be a liability under metabolic conditions that lead to elevated MGO/GSH ratios (*i.e.,* if GSH is being depleted and MGO is rising simultaneously), such as increased glycolytic flux or acute redox stress in specific tissues or cancer cells. Additionally, it is possible that the formation of any or all of the GSH-MICA adducts detected here are actively regulated through further enzymatic action or transport out of the cell, collectively regulating intracellular levels under basal conditions.

### Efflux Pump Protein MRP1 Actively Exports Glutathione-Derived MICA Metabolites

**Figure 4:**
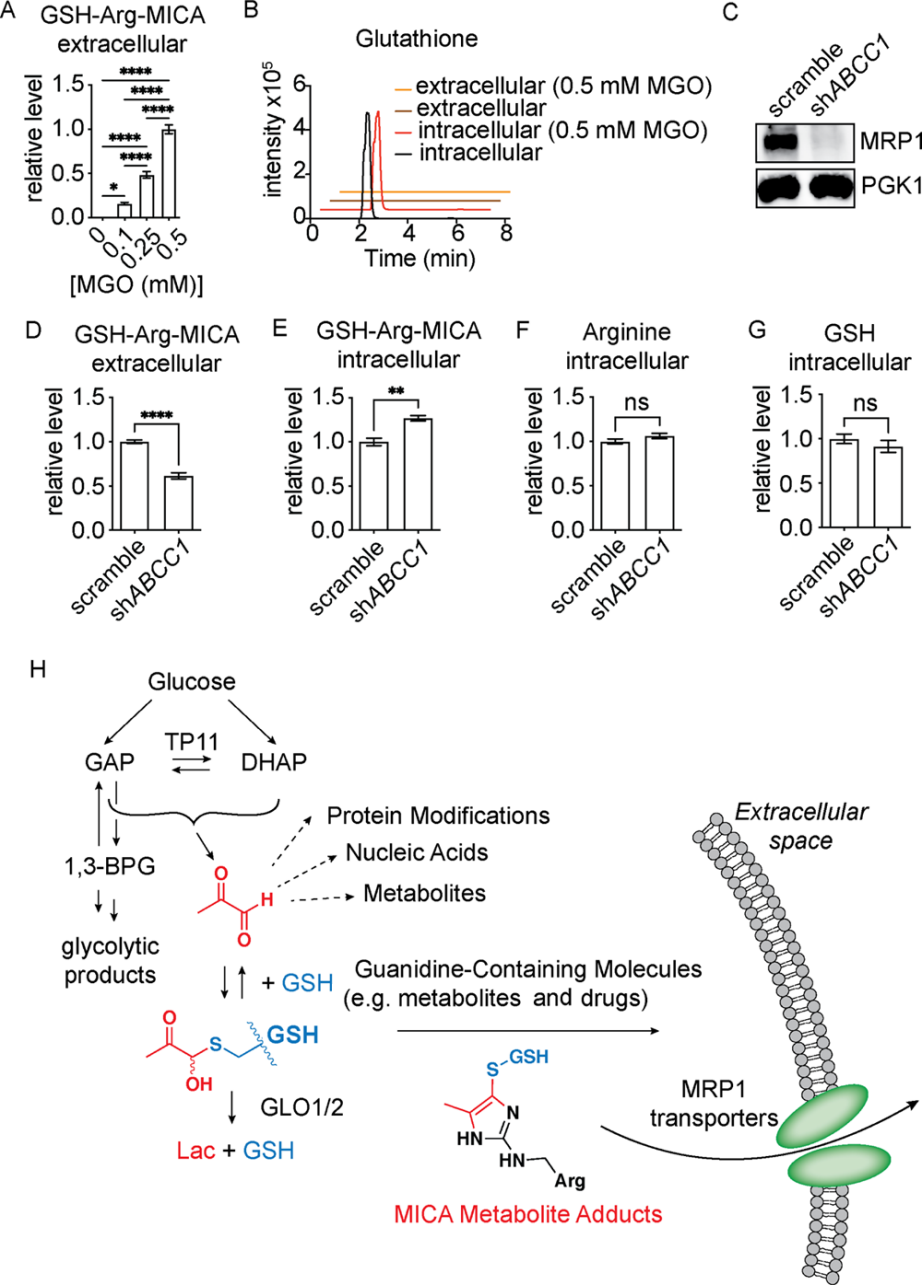
Glutathione-containing MICA metabolites are actively exported by the MRP1 transporter. (A) LC–MS/MS quantification of extracellular GSH-Arg-MICA from HeLa cells treated with indicated doses of MGO for 8 h. (B) Representative chromatograms of intra- and extracellular glutathione levels from HeLa cells treated for 8 h with indicated doses of MGO. (C) Western blot analysis of MRP1 and PGK1 levels in HeLa cells stably transduced with either sh*ABCC1* or control plasmid (scramble). (D–G) LC–MS/MS quantification of intra- and extracellular GSH-Arg-MICA, intracellular arginine, and intracellular glutathione in sh*ABCC1* or scramble HeLa cells treated with 0.5 mM MGO for 8 h. (H) Schematic depicting the deleterious interaction targets of glucose-derived MGO (dashed arrows) and protective capture of MGO by reduced glutathione for glyoxalase-dependent detoxification and, as shown in this work, parallel MICA metabolite formation and excretion from cells. Data plotted in (A) and (D–G) are mean with S.E.M. (*n* = 4 independent biological replicates). Statistical analysis in (A) is by ordinary ANOVA and (D–G) by unpaired Student’s *t*-test. *p < 0.05; **p < 0.01; ***p < 0.001; ****p < 0.0001.

While the GLO1/2 axis is the dominant MGO detoxification pathway in cells,^7,17^ there is no known enzyme that has been shown to unambiguously detoxify stable MGO modifications such as MG-H1 and MICA, either *via* enzymatic degradation or active export. Mirroring intracellular measurements, we observed dose-dependent buildup of extracellular GSH-Arg-MICA metabolite levels upon MGO treatment (Figure 4A). Since there was no detectable free glutathione in the media of either control- or MGO-treated cells (Figure 4B), this suggested that this metabolite may be actively exported from cells. A variety of glutathione conjugates of both endogenous metabolites and xenobiotic compounds have been shown to be actively exported by multidrug resistance-associated proteins, notably MRP1, encoded by the *ABCC1* gene.^28,29^ To test the hypothesis that MRP1 contributes to cellular detoxification of MICA cross-linked glutathione, we stably knocked down *ABCC1* in HeLa cells (*ABCC1*-KD) *via* short hairpin RNA (shRNA) (Figure 4C). When treated with MGO, *ABCC1*-KD cells had significantly higher intracellular levels and lower extracellular levels of the MICA crosslink of glutathione to arginine as compared to control scramble-KD cells (Figure 4D,E). This indicates that the MICA crosslink of glutathione to arginine is at least partially exported by MRP1, although other transporters or mechanisms of export for the MICA crosslink of arginine to glutathione likely exist. We also found that knockdown of *ABCC1* did not affect intracellular levels of glutathione or arginine, suggesting that the effect was not due to altered levels of the metabolite precursors (Figure 4F,G). We observed similar results for the GSH-Met-MICA metabolite levels inside and outside of cells treated with a combination of metformin and MGO (Figure S4A,B). Interestingly, although the intracellular levels of glutathione were again unchanged (Figure S4C), we did observe higher intracellular levels of metformin in the *ABCC1*-KD cells (Figure S4D). It is possible that MRP1 also actively exports metformin from cells, although, to our knowledge, this is yet to be demonstrated. These data suggest that under certain metabolic conditions, guanidine containing metabolites, drugs and proteins residues may react with methylglyoxal and glutathione to form stable MICA adducts. These metabolites, or perhaps the proteasomal degradation products of modified proteins, may subsequently be removed from the cell by active export by MRP1 and other proteins. Such a mechanism could operate alongside the dominant glyoxalase pathway, with both relying on initial GSH trapping of MGO prior to enzymatic and active export detoxification (Figure 4H).

This study aimed to help define the plausible reactivity landscape between MGO and biologically relevant thiols and guanidine containing molecules. In doing so, we have confirmed that significant intermolecular and intramolecular formation of mercaptomethylimidazole adducts on metabolites and proteins can occur *in vitro*, and some of these form in cells. Kinetic and thermodynamic studies reinforced the notion that local proximity of thiol/guanidine pairs accelerates formation of MICA adducts but that kinetically trapped hemithioacetals, for example, on surface cysteines, can go on to form MICA adducts with guanidine-containing molecules in solution. These data suggest that small-molecule MICA adducts may exist on native proteins and like other MGO adducts (*e.g.,* often cited as MG-H1 modifications) may alter protein functions and/or serve as disease biomarkers. Moreover, the kinetic trapping of methylglyoxal with thiol compounds and protein cysteines suggests that reversible modification of reactive cysteines in proteins might be a mechanism by which specific proteins could respond to changes in the methylglyoxal level and, by extension, changes in glucose metabolism and glycolytic flux. Future studies are warranted to define the landscape of thiol reactivity toward methylglyoxal throughout the proteome in order to identify functional regulation of protein activity by methylglyoxal either *via* MICA modification or through reversible hemithioacetal modification.

Beyond reversible and irreversible MGO modification of protein cysteines, these data show that MGO, GSH, arginine, and biguanide drugs can react to form several previously uncharacterized metabolites. The formation and regulated export of GSH-Arg-MICA and GSH-Met-MICA were particularly intriguing as our study demonstrates a novel route for the nonenzymatic trapping and removal of methylglyoxal from cells. This reactive capture and active export by MRP1 and perhaps other proteins may represent a parallel path to remove MGO-modified amino acids, cofactors, and free metabolites. Moreover, this nonenzymatic trapping and export mechanism appears to operate as a tandem metabolic branch adjacent to the glyoxalase enzymes, with each stemming from the common hemithioacetal conjugate of GSH and MGO (Figure 4H). While relative quantification of these metabolites was used in this study primarily to gauge kinetics and dose–response profiles, it is important that the absolute levels of GSH-MICA metabolites in specific cell types and contexts be determined in future work. These studies will help determine whether metabolites such as GSH-Arg-MICA have any functional role(s) or if additional routes of enzymatic or export pump-mediated detoxification exist. Alongside other work in this field, we posit that further characterization of the detoxification mechanisms of AGEs may provide insights into how cells prevent buildup of metabolite-derived cellular damage over physiologic timescales and in the myriad diseases associated with dysregulated glycolytic metabolism.^19,30−33^

## Methods

Fmoc-protected amino acids and resin were from Novabiochem. All other reagents were obtained from Sigma-Aldrich, and all bulk solvents were obtained from Thermo Fisher Scientific, unless otherwise stated.

### Methylglyoxal Synthesis and Quantification

High-purity MGO was prepared by acidic hydrolysis of MG-1,1-dimethylacetal, followed by fractional distillation and concentration quantified by the colorimetric assay, as previously reported.^34,35^ In brief, 6 mL of MG-1,1-dimethylacetal was added to 100 mL of 2.5% (v/v) sulfuric acid and refluxed for 1 h. The product was purified by fractional distillation under reduced pressure. The first fraction collected was discarded due to methanol impurity. To quantify MGO concentration, aliquots of the fractions were diluted with 50 mM sodium phosphate buffer pH 7.4 to be below an estimated 2 mM concentration and reacted with equal volume of 40 mM aminoguanidine in phosphate buffer for 5–6 h at 37 °C. Absorbance was measured at 320 nm and compared to a calibration curve generated by serial dilutions of 3-amino-1,2,4-triazine in phosphate buffer. MGO fractions were then diluted to 50 mM stock solutions using phosphate buffer, and pH was confirmed to be 7.4. MGO stocks were stored at −80 °C until use.

### Peptide Synthesis

The CRV2 peptide was synthesized using the standard solid-phase peptide synthesis technique with Fmoc-protected amino acids on MBHA rink amide resin. The peptide was cleaved in a solution of 94% trifluoroacetic acid, 2.5% triisopropyl silane, 2.5% H_2_O, and 1% β-mercaptoethanol. Peptides were purified *via* reverse-phase HPLC on an Agilent Zorbax SB-C18 column (250 × 9.4 mm, 5 μm).

### *In Vitro* Dose–Response MICA Formation Experiments

1 mM arginine or metformin-HCl, 1 mM *N*-acetylcysteine or glutathione (reduced), and 0, 0.1, 0.2, 0.5, 1, 2, 5, or 10 mM methylglyoxal were incubated in PBS at 37 °C for 24 h. For the CRV2 peptide, 1 mM peptide was used following the same reaction conditions. Reactions were diluted 1:2 with 0.1% trifluoroacetic acid in H_2_O and then frozen at −20 °C for later analysis.

### *In Vitro* Time-Course MICA Formation Experiments

Reactions were initiated with 1 mM arginine or metformin-HCl, 1 mM *N*-acetylcysteine or glutathione (reduced), and 1 mM methylglyoxal in PBS at 25 °C. For the CRV2 peptide, 1 mM peptide was used following the same reaction conditions. Reactions were sampled once an hour by an autosampler for LC–MS analysis starting at 0 min.

### Mass Spectrometric Quantification of *In Vitro* MICA Metabolite Experiments

All *in vitro* samples were analyzed on an Agilent 6540 Q-TOF MS/MS with 1290 UHPLC and 1260 nanoLC-Chip set to positive ion mode with a mass window of 50–1000 *m*/*z*. The capillary voltage was set to 3.5 kV. The drying gas temperature was 300 °C, flow rate = 8 L/min, and the nebulizer pressure was 35 psi. The fragmenter voltage was set to 150 V. Chromatography was performed with a Phenomenex Gemini C18 column (50 × 4.6 mm, 5 μm) at a flow rate of 0.4 mL/min. Mobile phase A (buffer A) was 0.1% trifluoroacetyl (TFA) in H_2_O, and mobile phase B (buffer B) was 0.1% TFA in CH_3_CN. The instrument was run at 0.4 mL/min with the following gradient: 0% buffer B (0–2 min); 0–30% buffer B (2–5 min); 30–100% buffer B (5–6 min); 100% buffer B (6–7 min); 100–0% buffer B (7–8 min); 0% buffer B (8–11 min). For peptide *in vitro* experiments, the following gradient was used: 0–60% buffer B (0–5 min); 60–100% buffer B (5–6 min); 100% buffer B (6–7 min); 100–0% buffer B (7–8 min); 0% buffer B (8–11 min). Relative metabolite abundance was quantified by the integrated peak area for each extracted ion chromatogram with a mass window of ±0.1 and normalized to the most abundant peak in a given time– or dose–response series.

### MGO Modification of BSA Proteomic Experiments

BSA (0.5 mg/mL) in PBS was incubated with 0.5 mM methylglyoxal and 0.5 mM arginine at 37 °C for 24 h. Following incubation, the BSA was dialyzed into fresh PBS 3 times using Amicon ultracentrifugal filter units with 30 kDa cutoff (Millipore). The protein was diluted to the original volume with PBS and supplemented with 1 mM MgCl_2_ using a 100 mM stock solution in water. Sequencing-grade trypsin (Thermo Fisher Scientific) was added at a 1:100 trypsin/protein ratio and digested overnight at 37 °C. Tryptic digests were desalted using 100 μL Pierce C18 tips (Thermo Fisher Scientific) according to the manufacturer’s protocol and then dried *via* a lyophilizer.

LC–MS/MS was performed with an Easy-nLC 1000 ultrahigh-pressure LC system (Thermo Fisher Scientific) using a PepMap RSLC C18 column (75 μm × 15 cm; 2 μm, 100 Å) heated to 45 °C. The LC system was coupled to a Q Exactive HF orbitrap and EASY-Spray nanosource (Thermo Fisher Scientific). Mobile phase A was composed of 0.1% formic acid in H_2_O, and mobile phase B was 0.1% formic acid in CH_3_CN. The instrument was run at 0.3 μL/min with the following gradient: 2% buffer B (0–5 min); 2–20% buffer B (5–45 min); 20–32% buffer B (45–55 min); 32–70% buffer B (55–56 min); 70% buffer B (56–59 min). MS/MS spectra were collected from 0 to 56 min using a data-dependent, top-10 ion setting with the following details: full MS scans were acquired at a resolution of 120,000, scan range of 375–1500 *m*/*z*, maximum IT of 60 ms, automatic gain control (AGC) target of 1 × 10^6^, and data collection in the profile mode. MS2 scans were performed by higher-energy C-trap dissociation (HCD) fragmentation with a resolution of 30,000, AGC target of 1 × 10^5^, maximum IT of 60 ms, normalized collision energy of 27, and data collection in the profile mode. The isolation window for precursor ions was set to 2.0 *m*/*z*. Peptides with a charge state of 1 and unassigned were excluded, and dynamic exclusion was set to 20 s. The S-lens RF level was set to 60 with a spray voltage value of 2.60 kV and an ionization chamber temperature of 300 °C. MS2 files were generated and searched using the ProLuCID algorithm in the Integrated Proteomics Pipeline (IP2) software platform. Data were searched using a concatenated target/decoy UniProt database of the BSA protein. Basic searches were performed with the following search parameters: HCD fragmentation method; monoisotopic precursor ions; high resolution mode (3 isotopic peaks); precursor mass range of 600–6000 and initial fragment tolerance of 600 ppm; enzyme cleavage specificity at C-terminal lysine and arginine residues with three missed cleavage sites permitted; two total differential modification sites per peptide, including oxidized methionine (+15.9949 M), MICA (+210.1116 C), MG-H1 (+54.0106 R), CEL (+72.0211 K), and CEA (+72.0211 R); primary scoring type by XCorr and secondary by Z-score; and minimum peptide length of six residues with a candidate peptide threshold of 500. Starting statistics were performed with a delta mass cutoff of 10 ppm with modstat and trypstat settings. False discovery rates were set to 1% at the peptide level. Reported modified peptides were required to meet the above criteria and were detected in at least two different experiments.

### Cell Culture

HeLa and HEK293T cells were purchased from ATCC and were propagated in RPMI 1640 medium supplemented with 10% fetal bovine serum and 1% penicillin/streptomycin (Gibco).

### Preparation of Samples for Metabolomic Experiments

Two million HeLa cells were plated and grown in 10 cm plates for 24 h prior to treatment with MGO, metformin, or combinations (in 5 mL media, for 8 h). The cells were collected by trypsinization, washed once with PBS, and resuspended in 300 μL of an 80:20 mixture of cold MeOH/H_2_O, and an internal standard (1 μL of 10 mM d3-serine) was added to the extraction solution. The mixture was sonicated for 10 s (1 s on/off cycles) followed by centrifugation at 16,000*g* and 4 °C for 10 min. The supernatant was collected and dried by SpeedVac. For extracellular metabolomics, 200 μL of the medium was collected and added to 800 μL of cold MeOH along with 3 μL of 10 mM d3-serine. The samples were mixed by vortexing and then centrifuged, supernatant collected, and dried by SpeedVac.

### Standards for QQQ

10 mM arginine or metformin-HCl, 10 mM cysteine or glutathione (reduced), and 10 mM methylglyoxal were incubated in PBS at 37 °C for 24 h. Reactions were diluted with 0.1% trifluoroacetic acid in H_2_O for analysis. Reaction mixtures were used in conjunction with Agilent MassHunter Optimizer software to develop MRM transitions and to determine elution times for metabolites.

### QQQ Metabolomics

Dried metabolome samples were resuspended in 45 μL of buffer A and clarified by centrifugation at 16,000*g* for 10 min. Extracellular samples were processed similarly but in a volume of in 200 μL. Targeted MS/MS analyses were performed on an Agilent triple quadrupole LC–MS/MS instrument (Agilent Technologies 6460 QQQ) set to positive ion mode. The capillary voltage was set to 4.0 kV. The drying gas temperature was 300 °C, flow rate = 5 L/min, and nebulizer pressure = 45 psi. The mass spectrometer was run in the MRM mode with delta EMV(+) set to 200. The MRM parameters are listed in extended data Table S1. Chromatography was performed with a Phenomenex Gemini C18 column (50 × 4.6 mm, 5 μm) at a flow rate of 0.4 mL/min. Buffers A and B, as described above, were used for all experiments. The instrument was run at 0.4 mL/min with the following gradient: 0% buffer B (0–3 min); 0–100% buffer B (3–10 min); 100% buffer B (10–11 min); 100–0% buffer B (11–12 min); 0% buffer B (12–15 min). Relative metabolite abundance was quantified by integrated peak area for the given MRM transition normalized to that of the internal standard.

### Generation of Stable shRNA Knockdown Cells

The *ABCC1* shRNA plasmid was generated by cloning forward and reverse primers (extended data Table S2) into the pLKO.1 puro plasmid backbone. The scramble plasmid refers to SHC002 (Sigma). Lentivirus was generated in HEK293T cells by transient transfection of the above vectors with pSPAX2 and pMD2.G packaging vectors (Addgene plasmids #11260 and #12259) using lipofectamine 2000. Viral supernatants were collected after 48 h of expression, passed through a 0.45 μm syringe filter, and supplemented with 8 μg/mL of polybrene (hexadimethrine bromide) before exposure to target cells. Selection was performed with 2 μg/mL puromycin.

### Western Blotting

The cells were washed twice with PBS, collected by scraping in radioimmunoprecipitation assay buffer with EDTA-free complete protease inhibitors (Roche), and sonicated on ice for 15 s (1 s on/off cycles). Insoluble debris was cleared by centrifugation at 16,000*g* and 4 °C for 15 min. The supernatant was diluted into 4× Laemmli buffer containing 100 mM βME, heated to 95 °C for 5 min, cooled to room temperature, resolved on a 10% sodium dodecyl sulfate-polyacrylamide gel electrophoresis gel, and transferred onto a nitrocellulose membrane by standard Western blotting methods. The membranes were blocked in 2% BSA in TBS + 0.1% Tween-20 (TBST). The antibodies used in this study include: anti-MRP1 (1:1000, #72202, Cell Signaling Technology) and anti-PGK1 (1:3000, sc-130335, Santa Cruz Biotechnology). Secondary donkey antirabbit 680 and donkey antimouse 800 (1:10,000, LiCor). The blots were imaged on a LiCor infrared scanner and images processed in ImageJ.

## Supporting Information Available

Structures showing MGO-derived modifications and model compounds used in the study; targeted fragmentation structures for mass spectrometry experiments; additional data related to sh*ABCC1* knock down experiments; modified BSA peptides detected in LC–MS/MS proteomics runs; MS/MS parameters for metabolomics; and primers used for cloning (PDF)
