# Supplementary material for: Methylglyoxal Forms Diverse Mercaptomethylimidazole Crosslinks with Thiol and Guanidine Pairs in Endogenous Metabolites and Proteins
Source: ACS Chem Biol. 2021 Sep 28;16(11):2453–61. doi: 10.1021/acschembio.1c00553 (PMC8609522; doi:10.1021/acschembio.1c00553)
Supplement: Supplementary file 1 — cb1c00553_si_001.pdf [file cb1c00553_si_001.pdf]

Supporting Information for:

Methylglyoxal forms diverse mercaptomethylimidazole crosslinks with thiol and guanidine pairs in endogenous metabolites and proteins

John S. Coukos & Raymond E. Moellering*
Department of Chemistry, The University of Chicago
929 E. 57th Street, Chicago, IL 60637

*e-mail correspondence to: rmoellering@uchicago.edu

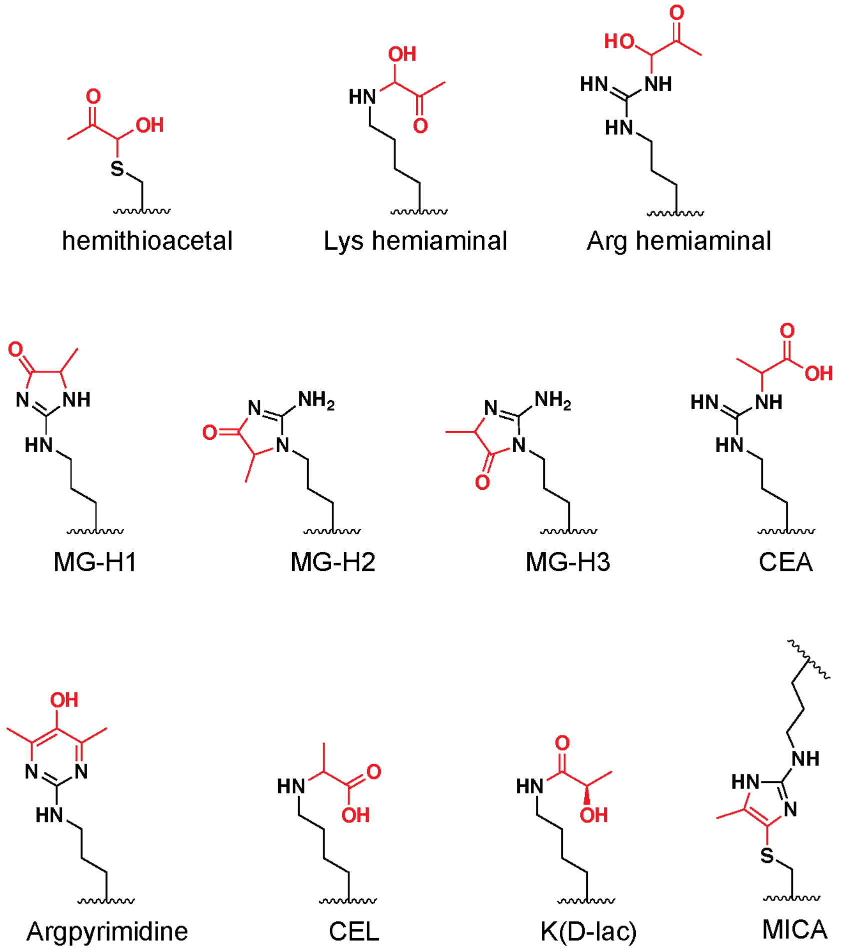

Figure S1: Known reversible and stable methylglyoxal-derived modifications.

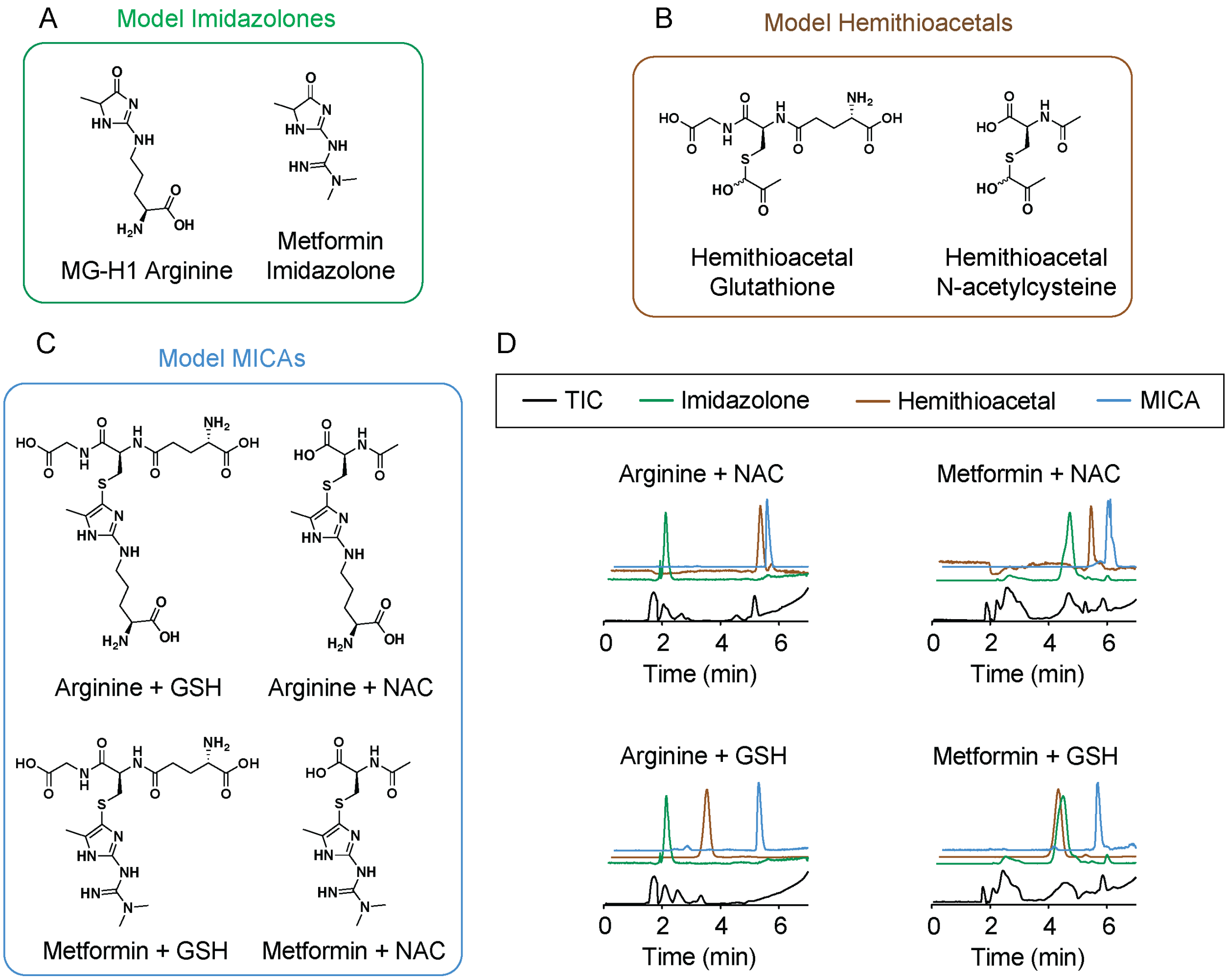

Figure S2: Methylglyoxal-derived modification of model imidazolones and thiols **(A)** Imidazolone derivatives of representative biologically relevant guanidine-containing small molecules. **(B)** Hemithioacetal derivatives of representative biologically relevant thiol-containing small molecules. **(C)** MICA crosslinks of representative biologically relevant guanidine and thiol containing small molecules. **(D)** Representative extracted ion chromatograms of the indicated products after 24-hour incubation of guanidine and thiol compounds (1 mM each) with 2 mM MGO at 37°C.

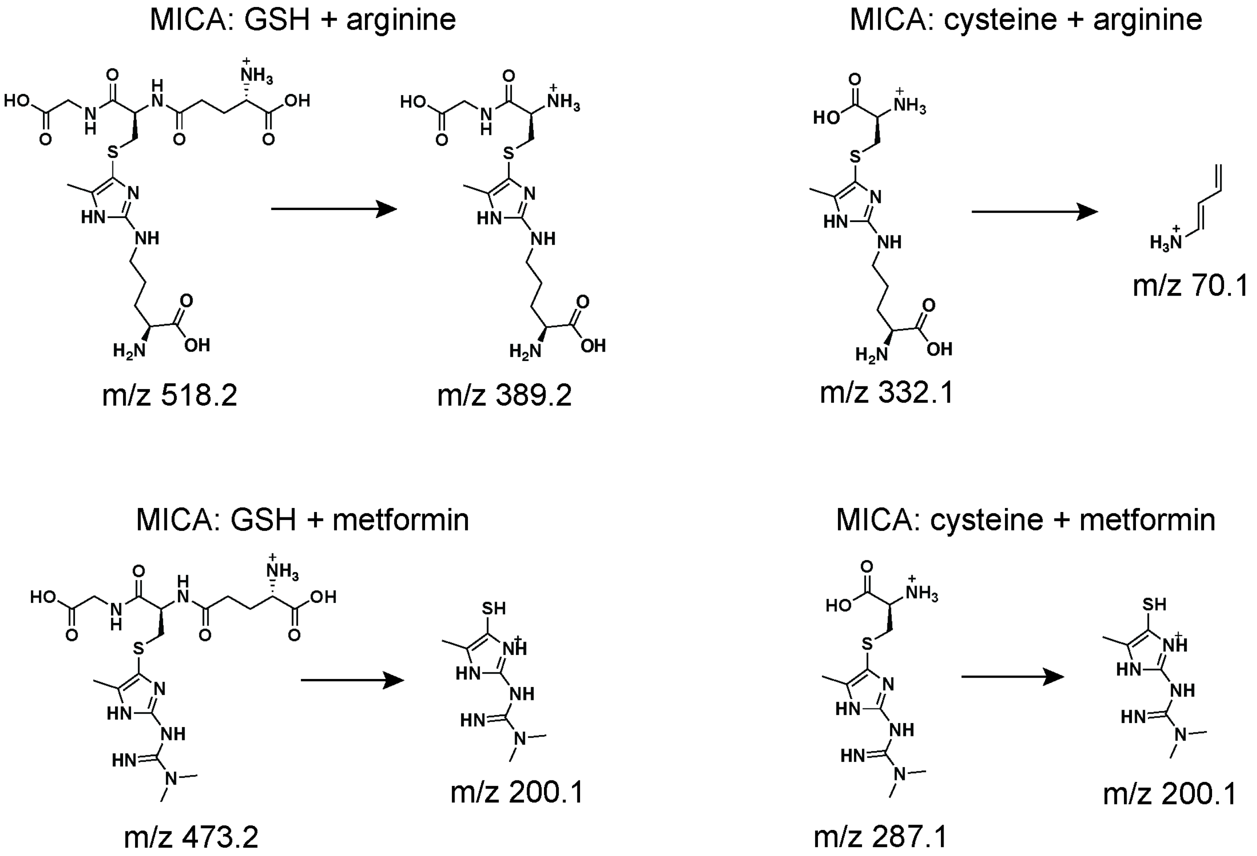

Figure S3: MICA MRM transitions. Chemical structures and m/z values for MRM transitions used to detect MICA crosslinks of glutathione or cysteine to arginine or metformin in this study.

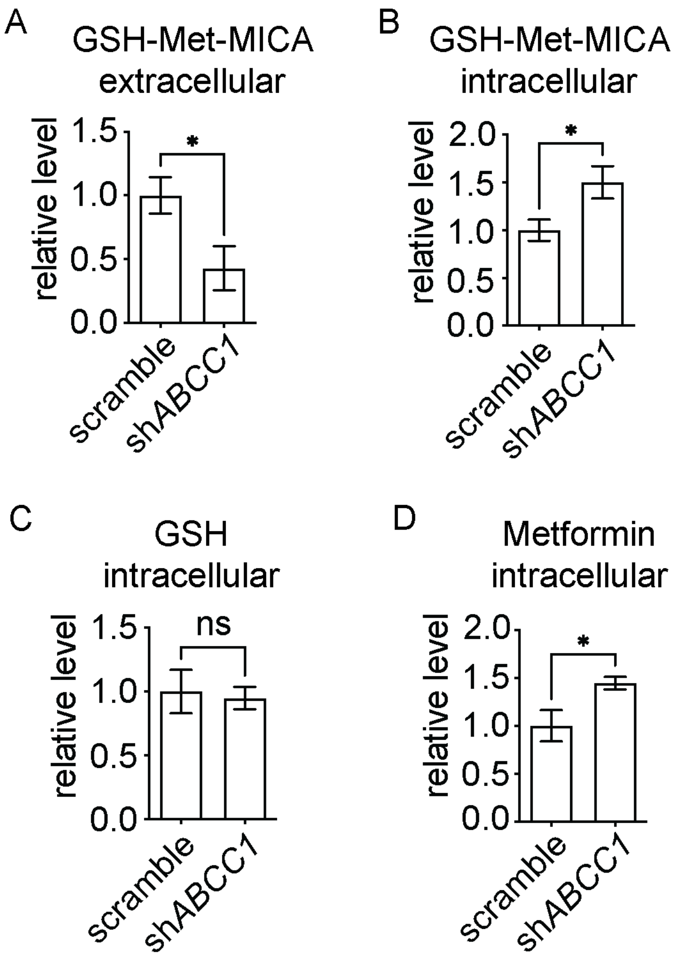

Figure S4: MICA crosslink of metformin to glutathione is exported by the MRP1 transporter. **(A-B)** LC-MS quantification of intra- and extracellular GSH-Met-MICA in *ABCC1*-KD or scramble-KD HeLa cells treated with MGO and metformin (1 mM each) for 8 hours. **(C-D)** LC-MS quantification of intracellular glutathione and metformin in *ABCC1*-KD or scramble-KD HeLa cells treated with MGO and metformin (1 mM each) for 8 hours. Data plotted in (A-D) are mean with S.E.M. from n = 4 independent biological replicates. Statistical analyses are by unpaired Student's t-test. *$p < 0.05$.

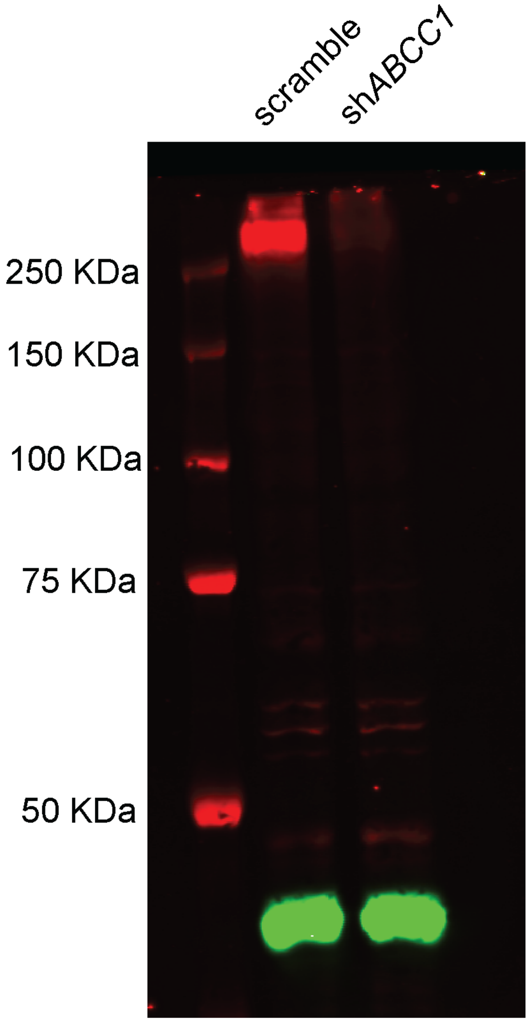

Figure S5: Representative *ABCC1* knockdown western blot: Full western blot corresponding to Figure 4C. MRP1 is in red, and PGK1 is in green.

Table S1: Post-translationally modified BSA peptides identified in this study

sequence	Residue	PTM	bio rep	Xcorr	DeltCN	ppm
K.GLVLIAFSQYLQQC(210.1116)PFDEHVK.L	C58	MICA	1,4	2.6531	0.5621	-0.3
K.FWGK(72.0211)YLYEIAR.R	K160	CEL	1,2,4	2.0445	0.6851	-0.1
R.K(72.0211)VPQVSTPTLVEVSRSLGK.V	K437	CEL	1,4	3.2704	0.6857	0
K.K(72.0211)QTALVELLK.H	K548	CEL	1,3,4	2.7941	0.7304	-0.3
K.YLYEIAR(54.0106)R.H	R167	MG-H1	1,2,3,4	2.3205	0.5527	1.2
K.YLYEIAR(72.0211)R.H	R167	CEA	2	1.5256	0.6159	-1.6
K.YLYEIAR(54.0106)R(54.0106)HPYFYAPELLYYANK.Y	R167,R168	MG-H1	1,4	3.4579	0.6163	0.8
K.YLYEIAR(72.0211)R(72.0211)HPYFYAPELLYYANK.Y	R167,R168	CEA	1	3.3189	0.5341	1.5
R.R(54.0106)HPYFYAPELLYYANK.Y	R168	MG-H1	1,2,3,4	5.3857	0.6989	0.7
R.R(72.0211)HPYFYAPELLYYANK.Y	R168	CEA	1,2,4	5.4732	0.6477	0.8
K.IETMR(54.0106)EK.V	R209	MG-H1	1,2,3	1.899	0.5293	-0.6
K.IETMR(72.0211)EK.V	R209	CEA	2	1.9485	0.3934	-0.4
K.FGER(54.0106)ALK.A	R232	MG-H1	1,2,3,4	2.3209	0.472	-0.8
K.FGER(72.0211)ALK.A	R232	CEA	2,3	2.4604	0.434	-1.7
K.AWSVAR(54.0106)LSQK.F	R241	MG-H1	1,2,3,4	3.0641	0.6314	0.4
K.AWSVAR(54.0106)LSQKFPK.A	R241	MG-H1	1,2	3.3429	0.5695	1.5
K.AWSVAR(72.0211)LSQK.F	R241	CEA	1,2,4	3.0052	0.508	-0.5
R.ALKAWSVAR(72.0211)LSQK.F	R241	CEA	1	2.2876	0.5612	2.2
K.SEIAHR(54.0106)FK.D	R34	MG-H1	3	1.5274	0.6714	-0.7
K.DAFLGSFLYEYSR(54.0106)R.H	R359	MG-H1	1,2,4	2.156	0.6684	0.9
R.R(54.0106)HPEYAVSVLLR.L	R360	MG-H1	1,2,4	4.123	0.7489	-0.4
R.R(72.0211)HPEYAVSVLLR.L	R360	CEA	1,2,3,4	3.0789	0.8051	-0.6
R.HPEYAVSVLLR(54.0106)LAK.E	R371	MG-H1	1	2.6334	0.6701	2.1
R.RHPEYAVSVLLR(54.0106)LAK.E	R371	MG-H1	1,2,4	4.4853	0.7124	1.9
R.RHPEYAVSVLLR(72.0211)LAK.E	R371	CEA	1,2	4.1838	0.7502	-0.6
K.LGEYGFQNALIVR(54.0106)YTR.K	R433	MG-H1	1,2,3,4	6.0906	0.7447	1.1
K.LGEYGFQNALIVR(54.0106)YTRKVPQVSTPTLVEVSR.S	R433	MG-H1	1,4	3.4388	0.4834	0.2
K.LGEYGFQNALIVR(72.0211)YTR.K	R433	CEA	1,2,3,4	3.2031	0.6117	0.3
K.LGEYGFQNALIVR(54.0106)YTR(54.0106)K.V	R433,R436	MG-H1	1,2,3,4	3.9823	0.5929	1.3
R.YTR(54.0106)KVPQVSTPTLVEVSR.S	R436	MG-H1	1	1.9318	0.5352	2.4
K.VPQVSTPTLVEVSR(54.0106)SLGK.V	R451	MG-H1	1,2,3,4	6.752	0.6969	-0.3
K.VPQVSTPTLVEVSR(72.0211)SLGK.V	R451	CEA	1,2,3,4	5.9312	0.6842	-0.5
R.KVPQVSTPTLVEVSR(54.0106)SLGK.V	R451	MG-H1	1,2,3,4	7.1652	0.6592	-0.2
R.KVPQVSTPTLVEVSR(72.0211)SLGK.V	R451	CEA	1,2,3,4	6.1075	0.8148	-0.3

Table S2: Acquisition parameters used for targeted metabolomics measurements.

Metabolite	Precursor ion	MS1 resolution	Product ion	MS2 resolution	Dwell	Fragmentor	Collision energy	Retention time (min)
Arginine	175. 1	Wide	70.1	Unit	25	106	25	1.7
MG-H1 arginine	229.1	Wide	70.1	Unit	100	110	29	1.9
Metformin	130.1	Wide	71.1	Unit	25	86	21	1.7
Glutathione	308.1	Wide	76.1	Unit	100	106	29	2.4
Cys-Arg-MICA	332.1	Wide	70.1	Unit	100	154	17	2.3
Cys-Met-MICA	287.1	Wide	200.1	Unit	100	184	25	5.8
GSH-Arg-MICA	518.2	Wide	389.2	Unit	100	192	17	5.8
GSH-Met-MICA	473.2	Wide	200.1	Unit	100	184	25	5.9
d_3-serine	109.07	Wide	63.1	Unit	100	40	13	1.7

Table S3: Primers for cloning shRNA knockdown plasmids.

shRNA	Primer (Forward)	Primer (Reverse)
ABCC1	CCGGCCTCTCAGTGTCTTACTCATTCTCGAGAATGAGTAAGACACTGAGAGGTTTTTG	AATTCAAAAACCTCTCAGTGTCTTACTCATTCTCGAGAATGAGTAAGACACTGAGAGG
